# Detection of atovaquone-proguanil resistance conferring mutations in *Plasmodium falciparum *cytochrome b gene in Luanda, Angola

**DOI:** 10.1186/1475-2875-5-30

**Published:** 2006-04-05

**Authors:** Sónia Pimentel, Fátima Nogueira, Carla Benchimol, Vatúsia Quinhentos, Joana Bom, Luís Varandas, Virgílio do Rosário, Luís Bernardino

**Affiliations:** 1 Centro de Malária e Outras Doenças Tropicais / IHMT / UNL, Lisbon, Portugal; 2 Hospital Pediátrico de Luanda, Luanda, Angola; 3 Unidade de Clínica das Doenças Tropicais / IHMT / UNL, Lisbon, Portugal

## Abstract

**Background:**

The fixed dose combination atovaquone-proguanil is a recently introduced antimalarial for treatment and prophylaxis of *Plasmodium falciparum *malaria. It is highly effective with a good tolerability profile and a convenient prophylactic regimen. Nevertheless, cases of treatment failure have already been reported, which have been associated to mutations in the cytochrome b gene of the *Plasmodium (pfcytb)*. The presence of atovaquone-proguanil *in vivo *resistance conferring mutations in *pfcytb *gene in Luanda, Angola, was investigated, in order to make recommendations on prescribing this antimalarial as prophylaxis for travellers.

**Methods:**

Two hundred and forty nine blood samples from children hospitalized at Luanda Pediatric Hospital for malaria were studied. The PCR-RFLP methodology was used in order to identify *pfcytb *wild type codon 268 and two point mutations: T802A and A803C.

**Results:**

All samples were identified as wild type for *pfcytb *gene at codon 268. In the studied population, no mutations associated to atovaquone-proguanil treatment failure were found. Prevalence of the studied mutations in the region was estimated to be less than 0.77% (99% significance level).

**Conclusion:**

Atovaquone-proguanil can be recommended for use by travellers to Luanda with expected high efficacy. This represents an improvement compared to other currently used prophylatic antimalarials in this region. However, it is imperative to continue surveillance.

## Background

In the past decades, international travel has increased and more than 125 million international travellers visit malaria endemic countries every year [1]. This has turned the attention of scientific and public health authorities to the problem of imported and introduced malaria. The leading cause of imported malaria is *Plasmodium falciparum *[2-6] and antibody reaction to the parasite circumsporozoite antigen among international travellers has shown a high prevalence of inoculation [7]. Infection is especially relevant in sub-Saharan Africa travellers [7].

Accordingly, the number of imported malaria cases has increased. There are approximately 30,000 cases of imported malaria notified per year in non-endemic countries with important morbidity and mortality [2-6,8]. Both these indices can be lowered by adequate prophylaxis. Indeed, most cases of malaria are due to non-compliance or inadequate prophylaxis [4,5,9]. It is, therefore, imperative to find a chemoprophylactic antimalarial with a good tolerability and safety profile as well as an attractive prophylactic regimen.

The fixed-dose combination of 250 mg atovaquone and 100 mg proguanil per tablet (Malarone^®^) has recently been introduced for treatment and prophylaxis of *P. falciparum *malaria. Based on its similarity to ubiquinol, atovaquone acts on *P. falciparum *cytochrome b (*pfcytb)*, affecting the parasite mitochondrial respiratory chain and collapsing the mitochondria membrane potential [10-12]. This leads to apoptosis [12]. In addition, the combination also blocks dihydroorotate dehydrogenase, inhibiting pyrimidine synthesis [11,13]. Proguanil enhances the ability of atovaquone to collapse the mitochondrial membrane by a mechanism still unexplained [14]. The combination effectively inhibits the development of the liver and blood stages of *P. falciparum *[15-17].

As recently reviewed atovaquone-proguanil is highly effective as prophylaxis for up to six months [18,19] with an improved tolerability profile, a more convenient prophylactic regimen and better compliance.

It is now recommended as a prophylactic alternative to mefloquine or doxycycline in chloroquine-resistant areas [20]. Nevertheless, in many countries it is considered the first line antimalarial for prophylaxis in areas of chloroquine resistance and for imported malaria treatment (including emergency treatment) [21,22].

There have been some reports of atovaquone-proguanil treatment failure in travellers, associated to *pfcytb *gene mutations, particularly at codon 268, namely T802A and A803C [23-27]. Epidemiologic surveillance of emerging resistance to this combination can be done by screening for these mutations.

The presence of atovaquone-proguanil *in vivo *resistance conferring mutations in *pfcytb *gene in Luanda, Angola, was investigated. This was done in order to estimate the prevalence of these mutations in this region and make recommendations on prescribing this antimalarial as prophylaxis for travellers.

## Methods

Infected blood samples were obtained from children younger than 12 years of age hospitalized at Luanda Pediatric Hospital, Angola, during the years of 2003/2004. Both the hospital and IHMT Ethics Committee approved all the applied protocols.

An estimated prevalence of mutation T802A and A803C of 0.77 and 0.96%, respectively, was considered for population size calculation [27]. For an expected prevalence of 0.77%, with a significance level of 99%, the size of the studied population should be at least 213 children (StatCal, EpiInfoVersion 6, CDC, Atlanta). Samples were individually spotted on Whatman no 4 filter paper, after microscopic confirmation of *P. falciparum *infection. Chelex DNA extraction was done according to established protocols [28]. A modified semi-nested PCR-RFLP method was carried out to search for polymorphisms in *pfcytb *(T802A and A803C) [24,29]. Primer sequences are presented in Table 1. All amplifications were performed in an I-Cycler-IQ, BIO-RAD, thermocycler. The first amplification using the "OD/OR" primers was designed to produce a 716 bp fragment in the following conditions: 92°C (three minutes) (1^st ^segment – 1 cycle); 92°C (30 seconds), 57°C (30 seconds), 72°C (one minute) (2^nd ^segment – 9 cycles); 92°C (30 seconds), 52°C (30 seconds) 72°C (one minute) (3^td ^segment – 34 cycles); 72°C (three minutes) (4^th ^segment – 1 cycle). This amplicon contained all the single point polymorphisms. Reagent concentrations were: 1 mM MgCl_2 _(Fermentas, Lithuania), 10 μM primers (MWG-Biotech AG, Germany), 1 mM dNTPs (Fermentas, Lithuania), 120 U *Taq *polymerase (Fermentas, Lithuania), 500 ng DNA template from field samples. Outer PCR product was further diluted (1:50) and 1 μL of this solution was used for the second amplification.

**Table 1:** Primer sequences used for the amplification of *pfcytb *codon 268.

Primer	Sequence
OD	5'-CGCAACAGGTGCTTCTCTTGT-3'
OR	5'-ACAGAATAATCTCTAGCACCAAAAATCAT-3'
WtR	5'-GGTTTACTTGGAACAGTTTTTAACAaTG-3'
802D	5'-GTTTATTTGGAATTATACCTTTATCACATCCTGATAATGCTATC-3'
802R	5'-TAAACCAGCTGGTTTACTTGGAACAGTTTTTAACATTGtt-3'
803D	5'-CCTGAATGGTACTTTCTACCAgTT-3'

Small case indicates base changes to provide digest sites for restriction enzymes to distinguish wild type from mutations at codon 268. O: outer PCR; D: direct primer; R: reverse primer; Wt: wild type.

In the second amplification (nested or semi-nested amplification), three different pairs of primers were used, namely OD/WtR; 802D/802R; 803D/OR to distinguish between the three polymorphisms. The following conditions were used for amplication: 92°C (three minutes) (1^st ^segment – 1 cycle); 92°C (30 seconds), 65°C (30 seconds), 72°C (one minute) (2^nd ^segment – 9 cycles); 92°C (30 seconds), 60°C (30 seconds) 72°C (one minute) (3td segment – 34 cycles); 72°C (three minutes) (4^th ^segment – 1 cycle). Reagent concentrations were the same as for the first amplification. The products of the second amplification were confirmed by electrophoresis in ethidium bromide-stained 3% agarose gel.

For RFLP analysis, 4 μL of each nested product was used, with 0.1 U of each enzyme and specific buffer in a 20 μL final volume, incubated overnight at 37°C. Table 2 summarizes primer pairs and restriction enzymes used for the identification of each polymorphism.

**Table 2:** Primer pairs and restriction enzymes used for the identification of each *pfcytb *codon 268 polymorphism.

Codon 268	Type	Primer pair	Product size (bp)	Restriction enzyme	Restriction pattern	Fragment length (bp)
	Outer PCR	OD ; OR	716			

TAT	Wild type	OD ; WtR	600	*Mph1103-I*	Cuts Wt, T802A	570 (+30)
AAT	T802A	802D; 802R	146	*Dra-I*	Cuts T802A	104 (+42)
TCT	A803C	803D; OR	173	*Cai-I*	Cuts A803C	148 (+25)

Wt: wild type; bp: base pairs; D: direct primer; R: reverse primer.

The laboratory-established clone K1 of *P. falciparum *(*pfcytb *wild type) was used as amplification reaction intrinsic control. *Pfmdr1 *gene from the 3D7 clone and *serca *gene from *Plasmodium chabaudi *have a cutting site for *Dra-I *and *Cai-I*, respectively. They were used as restriction reaction positive controls.

## Results

**Figure 1:**
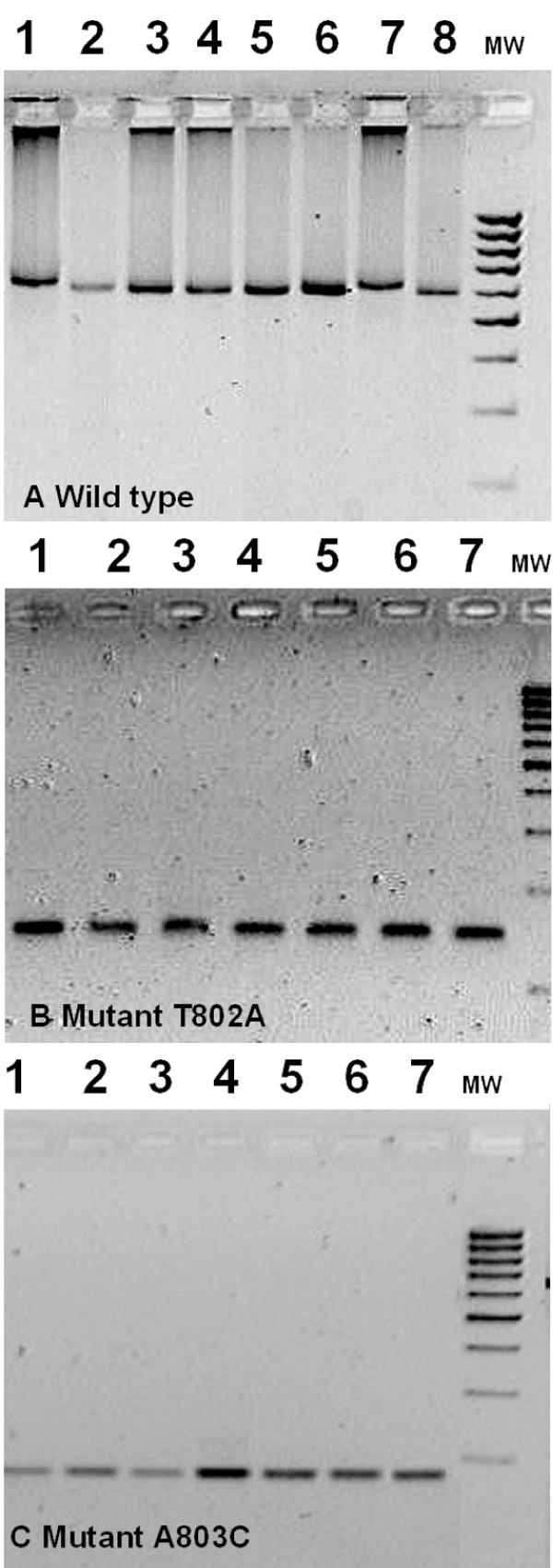
**Restriction digests for detection of *pfcytb *codon 268 mutations on field samples. **A. 600 bp amplification product with the primer pair for wild type (TAT) detection, digested by *Mph1103-I*; lane 1 and 7 – non-digested PCR product; lane 2 – K1 wild type; lanes 3–6, 8 field isolates. MWM – 100 bp molecular weight marker. B. 146 bp amplification product with the primer pair for mutation T802A (AAT) detection, digested by *Dra-I*; lane 1 and 7 – non-digested PCR product; lane 2 – K1 wild type; lanes 3–6 field isolates. MWM – 100 bp molecular weight marker. C.173 bp amplification product with the primer pair for mutation A803C (TCT) detection, digested by *Cai-I*; lane 1 and 7 – non-digested PCR product; lane 2 – K1 wild type; lanes 3–6 field isolates. MWM – 100 bp molecular weight marker.

Two hundred and forty nine blood samples were collected. DNA was successfully amplified from 224. Figure 1 illustrates the result of restriction of each of the second amplification products with the corresponding enzyme.

Products of the amplification with primers for the identification of wild type *pfcytb *were restricted with *Mph1103-I*. Clone K1 and all field samples were cut. None of the field samples amplified with primers for identification of T802A (AAT) and A803C (TCT) mutations was cut with either *Dra-I or Cai-I*, respectively. According to the enzyme restriction pattern, all samples were identified as *pfcytb *codon 268 wild type.

## Discussion

In the studied population, no mutations associated to atovaquone-proguanil treatment failure were found. Prevalence of these mutations in Luanda was estimated to be less than 0.77% with a 99% significance level. Therefore, Malarone^® ^can be recommended for use by travellers to this region with expected high efficacy. This represents an improvement in view of the known prevalence of resistance against other currently used prophylactic antimalarials.

This was the first work focusing on atovaquone-proguanil treatment failure associated mutations ever done in this area. It gives baseline information of the prevalence of these mutations in the region. After a more extensive atovaquone-proguanil introduction in the market, future studies will be important to monitor these numbers. Screening for the same mutations in Guinea Bissau, Zanzibar and Ghana [29,30] were also negative. Limited use of this antimalarial in endemic countries can explain the low prevalence of resistance-associated mutations. Restricted use by travellers will probably not increase pressure selection of mutants.

However, other cases of treatment failure, not associated to point mutations, have been described [27]. Study of prevalence of mutations in field samples, especially in treatment failure cases and travellers using the drug for prophylaxis, will help understanding the importance of these mutations in resistance. It is, therefore, imperative to continue surveillance.

## Authors' contributions

SP carried out the molecular genetic studies, participated in the design of the study and drafted the manuscript.

FN participated in the conception, design and coordination of the study, helped carrying out the molecular genetic studies, and helped to draft the manuscript.

CB was responsible for recruiting patients and collecting blood samples.

VQ was responsible for recruiting patients and collecting blood samples.

JB was responsible for DNA extraction from blood samples.

LV was responsible for conception, design and coordination of the study, and helped to draft the manuscript.

VR reviewed critically the manuscript and gave the final approval of the version to be published.

LB participated in the conception, design and coordination of the study.

All authors read and approved the final manuscript.

## References

[B1] World Tourism Organization http://www.world-tourism.org.

[B2] Muentener P, Schlagenhauf P, Steffen R (1999). Imported malaria (1985–95): trends and perspectives. Bull WHO.

[B3] World Health Organization The use of antimalarial drugs. Report of an informal consultation.

[B4] Jelinek T, Schulte C, Behrens R, Grobusch M, Coulaud J, Bisoffi Z, Matteelli A, Clerinx J, Corachán M, Puente S, Gjorup I, Harms G, Kollaritsch H, Kotlowski A, Björkmann A, Delmont JP, Knobloch J, Nielsen L, Cuadros J, Hatz C, Beran J, Schmid M, Schulze M, Lopez-Velez R, Fleischer K, Kapaun A, McWhinney P, Kern P, Atouguia J, Fry G, da Cunha S, Boecken G (2002). Imported falciparum malaria in Europe: sentinel surveillance data from the European Network on Surveillance of Imported Infectious Diseases. Clin Infect Dis.

[B5] Leder K, Black J, O'Brin D, Greenwood Z, Kain K, Brown G, Torresi J (2004). Malaria in travelers: a review of the Geosentinel Surveillance Network. Clin Infect Dis.

[B6] Shah S, Filler S, Causer L, Rowe A, Bloland P, Barber A, Roberts J, Desai M, Parise M, Steketee R (2004). Malaria Surveillance – United States, 2002. Surveil Summs.

[B7] Jelinek T, Blüml A, Löscher T, Nothdurft H (1993). Assessing the incidence of infection with *Plasmodium falciparum *among international travelers. Am J Trop Med Hyg.

[B8] Legros F, Danis M (1998). Surveillance of malaria in European Union countries. Eurosurveillance.

[B9] Kain KC, Harrington MA, Tennyson S, Keystone JS (1998). Imported malaria: prospective analysis of problems in diagnosis and management. Clin Infect Dis.

[B10] Fry M, Pudney M (1992). Site of action of the antimalarial hydroxynaphthoquinone, 2 – [trans – 4 (4' – chlorophenyl) cyclohexyl] – 3 – hydroxy-1, 4-naphthoquinone (566C80). Biochem Pharmacol.

[B11] Vaidya AB, Lashgari MS, Pologe LG, Morrisey J (1993). Structural features of *Plasmodium *cytohrome b that may underlie susceptibility to 8-aminoquinolines and hydroxynaphthoquinones. Mol Biochem Parasitol.

[B12] Srivastava IK, Rottenberg H, Vaidya AB (1997). Atovaquone, a broad spectrum antiparasitic drug, collapses mitochondrial membrane potential in malaria parasites. J Biol Chem.

[B13] Hudson AT (1993). Atovaquone – a novel broad-spectrum anti-infective drug. Parasitol Today.

[B14] Srivastava IK, Vaidya AB (1999). A mechanism for the synergistic antimalarial action of atovaquone and proguanil. Antimicrob Agents Chemother.

[B15] Shanks G, Gordon D, Klotz F, Aleman G, Oloo A, Sadie D, Scott T (1998). Efficacy and safety of atovaquone-proguanil as suppressive prophylaxis for *Plasmodium falciparum *malaria. Clin Infect Dis.

[B16] Shapiro TA, Ranasinha CD, Kumar N, Barditch-Crovo P (1999). Prophylatic activity of atovaquone against *Plasmodium falciparum *in humans. Am J Trop Med Hyg.

[B17] Berman JD, Nielsen R, Chulay JD, Dowler M, Kain K, Kester K, Williams J, Whelen A, Shmuklarsky (2001). Causal prophylactic efficacy of atovaquone-proguanil (Malarone) in a human challenge model. Trans R Soc Trop Med Hyg.

[B18] McKeage K, Scott L (2003). Atovaquone/Proguanil: A review of its use for the prophylaxis of *Plasmodium falciparum *malaria. Drugs.

[B19] Petersen E (2003). The safety of atovaquone/proguanil in long-term malaria prophylaxis of *Plasmodium falciparum *malaria. J Travel Med.

[B20] World Health Organization – International Travel and Health http://www.who.int/ith/en.

[B21] Kain K (2003). Current status and replies to frequently posed questions on atovaquone plus proguanil (Malarone^®^) for the prevention of malaria. Biodrugs.

[B22] Petersen E (2004). Malaria chemoprophylaxis: when should we use it and what are the options?. Expert Rev Anti Infect Ther.

[B23] Fivelman QL, Butcher GA, Adagu IS, Warhurst DC, Pasvol G (2002). Malarone treatment failure and in vitro confirmation of resistance of *Plasmodium falciparum *isolate from Lagos, Nigeria. Malar J.

[B24] Schwöbel B, Alifrangis M, Salanti A, Jelinek T (2003). Different mutation patterns of atovaquone resistance to *Plasmodium falciparum *in vitro and in vivo: rapid detection of codon 268 polymorphisms in the cytochrome b as potential in vivo resistance marker. Malar J.

[B25] Schwartz E, Bujanover S, Kain KC (2003). Genetic confirmation of atovaquone-proguanil resistant *Plasmodium falciparum *malaria acquired by a non-immune traveller to East Africa. Clin Infect Dis.

[B26] Färnert A, Lindberg J, Gil JP, Swedberg G, Berqvist Y, Thapar M, Lindegardh N, Berezcky S, Björkman A (2003). Evidence of *Plasmodium falciparum *malaria resistance to atovaquone and proguanil hydrochloride: case reports. BMJ.

[B27] Wichman O, Muehlberger N, Jelinek T, Alifrangis M, Peyerl-Hoffmann G, Mühlen M, Grobusch M, Gascon J, Matteelli A, Laferl H, Bisoffi Z, Ehrhardt S, Cuadros J, Hatz C, Gjørup I, McWhinney P, Beran J, Cunha S, Schulze M, Kollaritsch H, Kern P, Fry G, Richter J (2004). Screening for mutations related to atovaquone-proguanil resistance in treatment failures and other imported isolates of *Plasmodium falciparum *in Europe. J Infect Dis.

[B28] Singh B, Cox-Singh J, Miller AO, Abdullah MS, Snounou G, Rahman H (1996). Detection of malaria in Malaysia by nested polymerase chain reaction amplification of dried blood spots on filter papers. Trans R Soc Trop Med Hyg.

[B29] Gil JP, Nogueira F, Strömberg-Nörklit J, Lindberg J, Carrolo M, Casimiro C, Lopes D, Arez A, Cravo P, Rosário V (2003). Detection of atovaquone and Malarone^® ^resistance conferring mutations in *Plasmodium falciparum *cytochrome b gene (cytb). Mol Cel Probes.

[B30] Muehlen M, Schreiber J, Ehrhardt S, Otchwemah R, Jelinek T, Bienzle U, Mockenhaupt F (2004). Prevalence of mutations associated with resistance to atovaquone and to the antifolate effect of proguanil in *Plasmodium falciparum *isolates from northern Ghana. Trop Med Int Health.

